# Emirates consensus recommendations on cardiovascular risk management in type 2 diabetes

**DOI:** 10.3389/fendo.2024.1395630

**Published:** 2025-01-06

**Authors:** Hani Sabbour, Wael Almahmeed, Fatheya Alawadi, Abdullah Shehab, Abdulamjeed Al Zubaidi, Alaaeldin Bashier, Abdul Rauf Ghulam, Fauzia Rashid, Hosam Zaky, Hussien Heshmat Kassemn, Jamila Bin Adi, Juwairia Tahir, Khadija Hafidh, Mohammed Farghali, Mohamed Hassanien, James Januzzi

**Affiliations:** ^1^ Mediclinic Hospital, Abu Dhabi, United Arab Emirates; ^2^ Imperial College London Diabetes Center, Abu Dhabi, United Arab Emirates; ^3^ Warren Alpert School of Medicine, Brown University, Providence, RI, United States; ^4^ Cardiology, Cleveland Clinic, Abu Dhabi, United Arab Emirates; ^5^ Heart, Vascular and Thoracic Institute, Cleveland Clinic, Abu Dhabi, United Arab Emirates; ^6^ Endocrine Section, Dubai Hospital, Dubai Academic Health Corporation (DAHC), Dubai, United Arab Emirates; ^7^ College of Medicine, Mohammed Bin Rashid University of Medicine and Health Science (MBRU), Dubai, United Arab Emirates; ^8^ Cardiology Division, Mediclinic Hospitals, Al Ain, United Arab Emirates; ^9^ Burjeel Hospital, Abu Dhabi, United Arab Emirates; ^10^ Benefits Design and Strategic Purchasing Department, Healthcare, Abu Dhabi, United Arab Emirates; ^11^ Dubai Hospital, Dubai, United Arab Emirates; ^12^ Cardiology Department, Dubai Hospital, Dubai Academic Health Corporation (DAHC), Dubai, United Arab Emirates; ^13^ Cardiology Department, Zulekha Hospitals, Dubai, United Arab Emirates; ^14^ Cardiology Department, Cairo University, Cairo, Egypt; ^15^ The Emirates Society of Internal Medicine, Internal Medicine Rashid Hospital, Dubai Health Authority, Dubai, United Arab Emirates; ^16^ The Emirates Cardiac Society, Rashid Hospital, Dubai, United Arab Emirates; ^17^ Internal Medicine Department, Rashid Hospital, Dubai Academic Health Corporation (DAHC), Dubai, United Arab Emirates; ^18^ Medical Department, Dubai Medical College, Dubai, United Arab Emirates; ^19^ Massachusetts General Hospital, Harvard Medical School, Boston, MA, United States

**Keywords:** diabetes, heart failure, cardiovascular risks, biomarkers, UAE

## Abstract

**Background:**

The combination of cardiovascular disease and diabetes is a highly prevalent condition in the United Arab Emirates. Development and dissemination of evidence-based regional recommendations for optimal screening, treatment and referrals of people with diabetes and high cardiovascular risk is an important priority.

**Consensus panel:**

An expert panel of diabetologists, endocrinologists and cardiologists from the Emirates Cardiac Society and Emirates Diabetes and Endocrine Society as well as different entities in the UAE, discussed and reviewed evidence and also a consensus report from the American Diabetes Association to formulate contextualized recommendations that could be applied for optimal management of cardiovascular risk in people with diabetes in the UAE.

**Consensus findings:**

The combination of heart failure and other cardiovascular risks is a highly prevalent finding among people with diabetes in the United Arab Emirates. The causal inter-relationships between diabetes and heart failure are multifactorial and regular assessments of symptoms and steps for mitigation of risk factors are an important priority. The universal definition and classification of heart failure provides a useful framework for recommending optimal screening, treatment, and referral strategies to diabetic individuals at various stages of the cardiovascular continuum. Routine measurement (at least yearly) of natriuretic peptides and high-sensitivity troponins can help identify patients requiring cardiac imaging referrals. However, recommending routine measurements of natriuretic peptides and/or high-sensitivity troponins to all diabetic individuals must balance clinical judgment and cost implications. While SGLT2i must be an important part of the standard of care, insulin, GLP1 receptor agonists and/or metformin can be useful for additional glycemic control.

**Conclusion:**

The consensus panel hopes that the recommendations presented herein can offer guidance for optimal screening, treatment and referral of people with a concomitance of diabetes and high cardiovascular risk in the United Arab Emirates.

## 1 Introduction

A recent multidisciplinary in consensus statement by Pop-Busui et al. highlighted the under- appreciation of heart failure as a complication of diabetes (1). This consensus report from the American Diabetes Association, which has been endorsed by the American College of Cardiology presents several best-practice recommendations for screening, diagnosis and referral of people with a combination of diabetes and heart failure risk (1). Consensus statements differ from clinical practice guidelines and do not necessarily need grading of evidence (2). Consensus statements deal more with “what to do” kind of considerations rather than “why to do” considerations (2). Thus, very similar to the ADA consensus report, we propose this as a practical guide to the application of biomarker-based detection of complications of diabetes from a panel composed of the UAE consensus group of expert authors from multiple specialties including diabetologists, endocrinologists, cardiologists, internal medicine specialists, nephrologists, family medicine specialist and representatives of both health care regulators and insurance payors and the emirates medical association. Thus, the ADA consensus report must be viewed as a set of “what to do” recommendations derived through a consensus between a multidisciplinary group of experts.

Adapting evidence-based recommendations to the contextual specificities of a geographical region is a key aspect of evidence-based medicine (3, 4). Such Contextualization helps to ensure optimal and effective dissemination of evidence-based best practices in a region sensitive manner. The consensus statement presented herein has been synthesized by an expert multidisciplinary group of expert authors from multiple specialties including diabetologists, endocrinologists, cardiologists, internal medicine specialists, family medicine specialists, nephrologists, and representatives of both healthcare regulators and insurance payors and the Emirates Medical Association (from the Emirates Cardiac Society and Emirates Diabetes and Endocrine Society and Emirates Family Medicine Association) For optimal management (screening, diagnosis, and referral) of heart failure in people with diabetes in the Emirates region.

**Figure 1:**
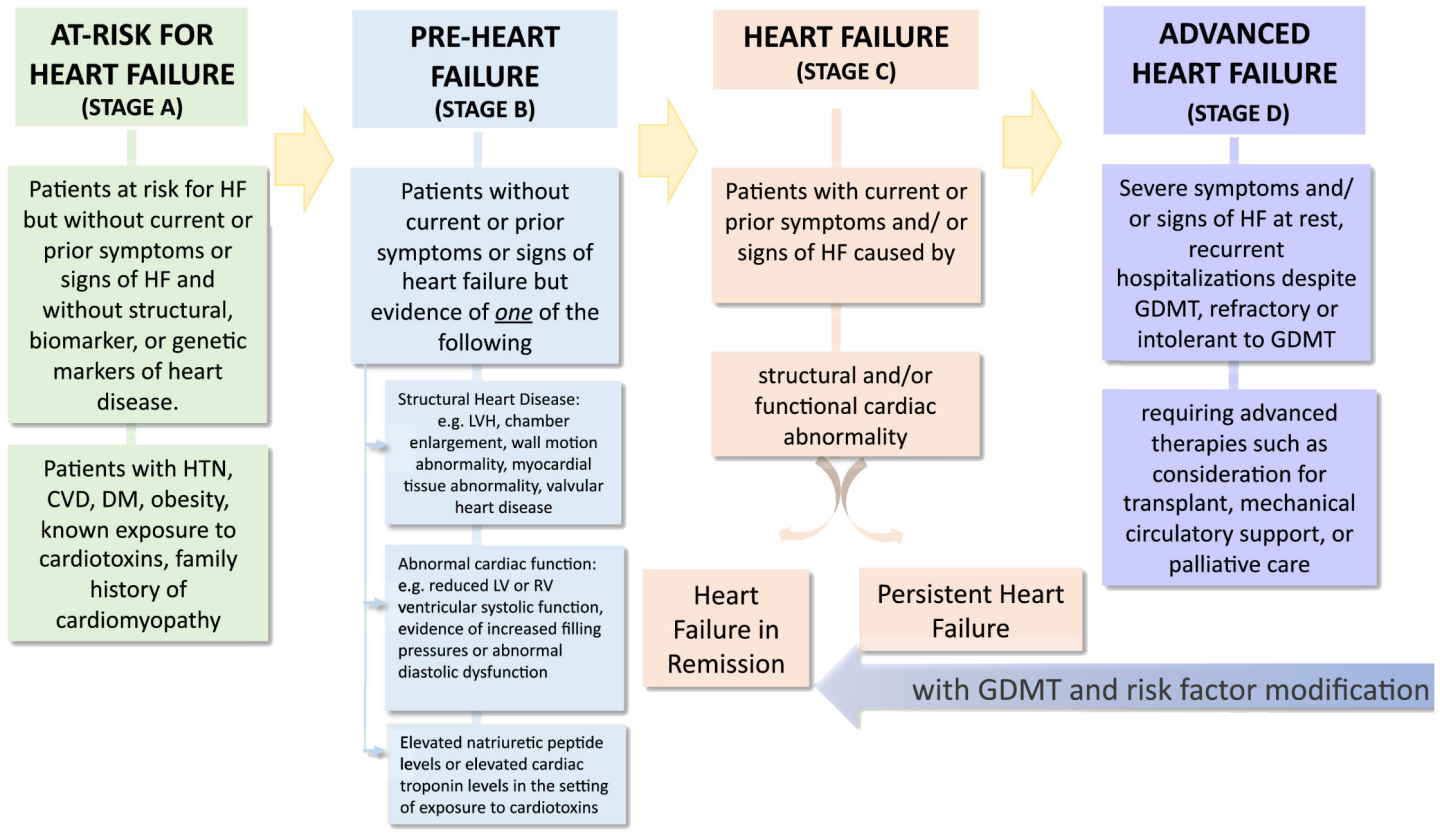
Reprinted with permission from / Adapted with permission from Universal Definition and Classification of Heart Failure A Report of the Heart Failure Society of America, Heart Failure Association of the European Society of Cardiology, Japanese Heart Failure Society and Writing Committee of the Universal Definition of Heart Failure by Biykem Bozkurt, Andrew JS Coats, Hiroyuki Tsutsui, Magdy Abdelhamid, Stamatis Adamopoulos, Nancy Albert, Stefan D. Anker, John Atherton, Michael Böhm, Javed Butler, Mark H. Drazner, G. Michael Felker, Gerasimos Filippatos, Gregg C. Fonarow, Mona Fiuzat et al., licensed under 5903040821098, Elsevier.

The Heart Failure Society of America, Heart Failure Association of the European Society of Cardiology and Japanese Heart Failure Society have together developed a Universal Definition and Classification of Heart Failure (**Figure 1**). Adapted from Bozkurt B. (5). This definition reduces the emphasis of ejection fraction and focuses on the diagnosis, prevention, early-stage disease and symptomatic stages (5). Thus, it provides a useful framework for individualizing screening, diagnosis and referral-focused recommendations and best practices according to the stage of heart failure risk in people with diabetes. Overall, the recommendations provided herein is an effort to offer an expert position statement for adapting the recommendations of the ADA consensus *to* the specific context of the United Arab Emirates.

## 2 Methods

An expert multidisciplinary group of cardiologists, diabetologists, and endocrinologists participated in a two-part meeting held virtually in 2022. It should be noted that the ESC guidance in 2019 recommended an algorithm for the diagnosis of HF in the primary care setting since this is a very common undiagnosed complications of Type 2 DM; however, this wasn’t widely adopted (6, 7).

In the first meeting, the multidisciplinary group reviewed the ADA consensus. Furthermore, 5 working groups were formed to review evidence for 5 separate topics. In the second meeting, the expert multidisciplinary group discussed this evidence to synthesize consensus-based recommendations for The management of cardiovascular risks in type 2 diabetes in the United Arab Emirates.

## 3 Consensus statements

### 3.1 Diabetes and conventional cardiovascular risk in the United Arab Emirates

The Association of diabetes and conventional risk factors leads to a significantly increased risk of cardiovascular events. It is recognized that if a diabetic patient has a single risk factor such as hypertension, obesity, dyslipidemia. they are considered high risk for cardiovascular events. In addition, diabetic patients with target organ damage as left ventricular hypertrophy or renal disease or albuminuria or retinopathy are at very high risk. Diabetics with multiple risk factors including any of the following: obesity, hypertension, hyperlipidemia, are at very high cardiovascular risk and this has been recognized in the guidelines from the ESC 2019. There are additional risk enhancers in patients with diabetes that are listed in the tables below (**Boxes 1**-**3**), which increases the risk of diabetic patients including an abnormal API and other biomarkers of atherosclerosis such as lipoprotein A and C-reactive protein. In the diabetic patients in the UAE additional testing has been recommended at an earlier age based on the UAE diabetes guidelines and the UAE cardiometabolic guidelines. Management of hypertension in diabetic patients is of paramount importance since it increases the risk of cardiovascular events significantly the recent American Heart Association ACC guidelines have lowered the threshold of treatment of hypertension in diabetes to encompass more treatment in a larger component of patients. It is well recognized that hypertension and coronary artery disease are major co-morbidities in diabetes and that Accelerated atherosclerosis is responsible for increased mortality and myocardial infarctions in diabetic patients. However, the less well-recognized complication is heart failure which will be expanded on in the next section (8).

Box 1 **Consensus statement – Epidemiological considerations.**

• **The concomitant burden of diabetes and heart failure is high in the United Arab Emirates**
**•** Steps to enrich currently available epidemiological data from the United Arab Emirates are recommended.
 ○ Registries and observational studies must include measures for assessing the concomitance and causal inter-relationships between diabetes and heart failure in a manner relevant to context of United Arab Emirates.
**• The high concomitance of diabetes and heart failure in the United Arab Emirates needs implementable action for**
 ○ Risk factor mitigation as a preventive strategy
 ○ Risk factor assessment for optimal referral for multidisciplinary interventions
 ○ Optimal screening for new-onset diabetes in people with heart failure
 ○ Optimal screening for undiagnosed heart failure in people with diabetes

Box 2 **Consensus statement – Cardio renal Complications in diabetes.**

• The diabetes cardio-renal spectrum presents an epidemiologically and pathophysiological interrelated “triple burden.”
• Studies to assess the impact of this triple burden in the United Arab Emirates is strongly recommended.
• Based on current evidence, the use of SGLT2 inhibitors are essential organ-protective and life- saving therapies in the standard of care in people with diabetes who are at the risk of cardiorenal syndromes.

Box 3 **Consensus statement – Clinical risk assessment.**

**•** Optimal clinical risk assessment is recommended in people with diabetes.
• Annual biomarker measurement (see next section) is recommended if the clinical risk factors are identified in people with diabetes.
• Referral to cardiology specialists if biomarkers identify CV disease.
• It is recommended that presence of these clinical risk factors and/or biomarkers must warrant considerations for change in treatment to drugs with established cardiovascular benefits

Box 4 **Consensus statement – Biomarkers and Risk Detection.**

• We recommend annual screening for all diabetic adults with natriuretic peptides.
• We also recommend measuring natriuretic peptide levels in diabetics who complain of dyspnea, fatigue, lower limb swelling, or chest pain.
• It is cost-effective, to utilize NT-proBNP and is recommended prior to echocardiography to rule out LV dysfunction in diabetic patients.
• In selected high-risk patients, there is additional prognostic information by testing C-reactive protein and high-sensitivity troponin in asymptomatic diabetic patients.
• Key symptoms that should be asked about during every visit include exertional fatigue, inability to go up one flight of stairs (NYHA functional class-II), lower limb edema, and exertional palpitation. In this situation, NT-proBNP and an ECG should be obtained with a fast-track referral to a cardiologist (99).

### 3.2 Epidemiology of diabetes and heart failure in the United Arab Emirates

Cardiovascular disease is the most common cause of mortality in people with diabetes and several studies have documented the high prevalence of cardiovascular risk factors in people with diabetes (9). Early identification and mitigation of these risk factors, especially the modifiable ones are essential health care priorities. In the last 50 years, UAE has witnessed significant economic growth, along with an increase in per-capita income and life expectancies (10). The lifestyle change associated with this economic growth has also led to increases in several modifiable risk factors such as a sedentary lifestyle, obesity, diabetes, hypertension and cardiovascular disease (10). A recent systematic review by Razzak et al. indicates that the prevalence of diabetes in the United Arab Emirates ranges from 0.87% to 33.3% (11). According to another report from Al Awadi et al., the prevalence of diabetes in the Emirates region was about 16.3% (12). These estimates underscore a high prevalence of diabetes. On the other hand, there is a relative paucity of heart failure data in the United Arab Emirates. However, according to a 2018 survey by AlHabeeb et al, an estimated 93,865 patients were receiving treatment for heart failure in the United Arab Emirates (13). Furthermore, the results of the Gulf CARE Registry indicate that heart failure patients from the Emirates region are about 10 years younger as compared to their age-matched Western population and about 50% of patients have diabetes (14). Overall, these data indicate that along with a high burden of diabetes, a substantial proportion of heart failure patients in the United Arab Emirates have diabetes.

Several studies have demonstrated the elevated risk of heart failure in people with diabetes. Results of the Framingham Heart Study indicate a 2-fold higher risk of heart failure in men with diabetes and a 4-fold higher increase in women with diabetes (9). A retrospective study by Nichols et al. indicates that diabetic individuals in the younger age groups may be particularly susceptible to the elevated risks of congestive heart failure (15). Shindler et al. in their report highlighted that diabetes is an independent predictor of outcomes in people with symptomatic heart failure asymptomatic left ventricular dysfunction (16).

Furthermore, the duration of diabetes and poor glycemic control are known to be risk-elevation factors for heart failure in people with diabetes (17, 18). Presence of comorbidities are also known modulators of the association between diabetes and heart failure (18, 19). Presence of macrovascular and microvascular complications of diabetes and higher levels of NT-proBNP (N-terminal pro-B-type natriuretic peptide) are known to increase the risk of incident heart failure (20–25).

Data from several studies also demonstrate the diabetogenic effects of heart failure. Paolillo et al., in their article highlighted the bidirectional association between heart failure and diabetes (26). In this study, insulin resistance was noted in about 63% of patients with severe-to-moderate heart failure (26). Heart failure also is known to aggravate the incidence of new onset diabetes. Data from the EMPHASIS-HF trial and the CHARM program indicate that the incidence of new-onset diabetes in heart failure patients could be in the range of about 21-28 per 1000 person-years, respectively (27, 28). Results of these studies indicate that factors such as elevated BMI, waist circumference, smoking along with abnormal glucose, HbA1c and systolic blood pressure aggravate the diabetogenic effects of heart failure (27, 28).

The risks of mortality, increased hospitalization, re-hospitalization and impaired quality-of-life are known to be aggravated by the bidirectional association of diabetes and heart failure places a significant burden on health outcomes and healthcare utilization (29).

Overall, available Emirates-specific data indicates that the prevalence and incidence of concomitant diabetes and heart failure is noticeably high in the United Arab Emirates (10–14). When viewed along with the data from other international studies presented above, it appears that several research and clinical recommendations need prospective implementation to attenuate the impact of diabetes and heart failure concomitance in the United Arab Emirates.

### 3.3 Cardio-renal complications in diabetes

A number of studies have documented the vicious epidemiological and pathophysiological inter- relationships between diabetes, heart failure, and chronic kidney disease. In a recent study, Schechter et al. note that the prevalence of cardiorenal syndrome (CRS) was 2.3% in patients with type 2 diabetes as compared to 0.4% in the entire population (N= 1,389,604; 52.2% females and 47.8% males) (30). This study further noted the prominence of type 2 diabetes among younger subjects within the diabetes-cardio-renal spectrum, which was gradually replaced by HF and eGFR < 60 mL/min/1.73m2 with increasing age (30). Ronco et al. describe five subtypes of cardiorenal syndrome, wherein type 1 and 2 cardiorenal syndrome occur because of the cardiac conditions impacting the kidneys, type 3 and 4 occur when renal conditions affect the heart. Type 5 CRS occurs when systemic conditions impact the heart and kidneys concurrently (31). Banerjee et al. in their study indicate that diabetes is strongly associated with Type 2 CRS (adjusted OR: 2.25 (CI 1.56-3.23, p < 0.001) (32). Being a systemic condition, diabetes is also known to be causally associated with type 4 and type 5 cardiorenal syndrome (32–34). Several interrelated mechanisms are known to underlie the manifestations of cardiorenal syndrome. Compensatory activation of RAAS (renin-angiotensin-aldosterone system), cardiac hypertrophy, and myocardial fibrosis along with inflammation, oxidative stress, and endothelial dysfunction are known to underlie pathophysiological mechanisms contributing to the bidirectional association between heart and kidney dysfunction (32, 33). Almost all of these pathophysiological factors also contribute to a “diabetic heart”, also known as diabetic cardiomyopathy or metabolic inflammatory cardiomyopathy.

Dei Cas et al. in their review indicate that poor outcomes in people with a concomitance of diabetes and heart failure as compared to those without diabetes could be explained by diverse metabolic and neurohormonal abnormalities (35). While the pathophysiological link between diabetes and heart failure is confounded by hypertension, microvascular dysfunction, and autonomic neuropathy, several mechanistic associations at systemic, cardiac, and cellular/molecular levels explain different aspects of myocardial dysfunction in people with diabetes (36).

It is relatively well-established that hyperglycemia and hyperinsulinemia both promote vascular smooth muscle cell proliferation and inflammation and aggravate atherosclerotic processes (37–42). The aggravating association of diabetes mellitus with atherogenic dyslipidemia and endothelial dysfunction are all known to adversely impact thrombosis, inflammation, and coronary plaque ulceration (43–45). The resulting ischemic or infarct-related consequences may underlie the heart failure and other structural heart abnormalities seen in people with diabetes. In fact, diastolic dysfunction due to left ventricular hypertrophy is seen in a majority of patients with diabetes (46–48). Levelt et al. note that cardiac steatosis may contribute to concentric remodeling and contractile dysfunction of the left ventricles in diabetes (49).

The impact of hyperglycemia on cardiac health can be explained by the effects of the former on formation of advanced glycation end products, which are implicated in collagen cross-linking, and consequent increases in myocardial fibrosis (50–52).

Furthermore, the effects of hyperglycemia on the activation of the Renin Angiotensin Aldosterone System (RAAS) and the resultant cardiac hypertrophy and exacerbation of diastolic dysfunction have been implicated in the pathological association between diabetes and heart failure (53).

Several studies have highlighted the significance of sodium–glucose cotransporter-2 (SGLT2) inhibition in attenuating the impact of cardio-renal syndrome (54, 55). While SGLT2 inhibition offers cardiovascular protection by reducing cardiac workload, blood pressure, and body weight, it offers additional protective effects on kidney by reducing albuminuria and hypoxic stress, and by restoring tubuloglomerular feedback (34).

Overall, available data indicates that diabetes and cardiorenal syndrome forms a burdensome “triple threat” with demonstrable epidemiological and pathophysiological inter-relationships at systemic and cellular levels (32, 34). While current evidence for SGLT2 inhibition is promising enough to attenuate the impact of this “triple threat”, accruing evidence from ongoing studies should refine our understanding on the place of SGLT2 inhibition in the management of diabetes and its cardio-renal complications (34).

### 3.4 Clinical risk assessment in T2D-clinical variables predicting heart failure

Zhou et al. in their systematic review point out that the main risk factors of heart failure in the type 2 diabetic population were age, hemoglobin A1c (HbA1c), coronary heart disease, hypertension, microalbuminuria and obesity (56). Yancy et al. in their article draw attention to the fact that several recent guidelines emphasize the need for optimal preventive and clinical management strategies for attenuating these risk factors to counter incident heart failure in people with diabetes (57). Although several models incorporating these risk factors have been developed and reported for predicting prognosis and outcomes of heart failure in people with diabetes, they lack clinical validation (58–60). Nevertheless, data from several randomized clinical trials and observational studies indicate that mitigating risk factors alleviate the burden of adverse cardiac outcomes, especially with atherosclerotic heart disease and stroke (61). However, until recently there has been a general paucity of data for specifically characterizing the impact of risk factor mitigation on incident heart failure and its outcomes. Data from the Swedish National Diabetes Register indicates that the rate of hospitalization for heart failure remains obstinately high despite optimal control of conventional risk factors (62). However, results of this registry indicate that the presence of atrial fibrillation, higher body-mass index, a low estimated glomerular filtration rate, and high glycated hemoglobin level are strong predictors for hospitalization with heart failure (62). A review of currently available evidence on the clinical risk factors underlying the concomitance of diabetes with heart failure is presented below.

A meta-analysis by Ohkuma et al. highlights that the gender disparity for heart failure among people with diabetes is skewed more towards women as compared to men (63). As per the results of this meta-analysis, the pooled multiple-adjusted relative risk for heart failure was 5.15 (95% CI 3.43, 7.74) in women and 3.47 (2.57, 4.69) in men with type 1 diabetes (63). Furthermore, the relative risk for heart failure was 1.95 (1.70, 2.22) in women and 1.74 (1.55, 1.95) in men with type 2 diabetes (63). Another study by Chadalavada et al. indicates that people with diabetes as compared to non-diabetic individuals had a twice as high risk of incident heart failure and mortality (64). Furthermore, in this study females with type 1 and type 2 diabetes had a 22% elevated risk of heart failure as compared to men (hazard ratio: 2.2 (95% CI: 1.9-2.5) vs. 1.8 (1.7-2.0) (63). Available evidence indicates that this gender-disparity could be due to a higher incidence of coronary heart disease, poor control of hypertension and glycemic control and a greater degree of endothelial dysfunction (65–69).

Concerning diabetes-related risk factors, it appears that both the level of glycemic control and duration of diabetes seem to have a significant effect on incident heart failure (70, 71). According to data from the Framingham Heart Study, each 10-year increase in the duration of diabetes is associated with a 25% increase in risk of cardiovascular events including hospitalization for heart failure (multivariable-adjusted hazard ratio: 1.25 (95% CI, 0.99– 1.57) (72). In the ARIC study, Echouffo-Tcheugui et al. report that each 5-year increase in the diabetes duration was associated with a 17% (95% CI: 11-22) relative increase in HF risk (18). Results of the UK Prospective Diabetes study demonstrated a 16% reduction in the risk of heart failure associated with a 1% reduction in glycated hemoglobin, thereby highlighting those patients with nonoptimal glycemic control have an increased risk of heart failure events (73). Results of a meta-analysis by Aune et al. indicate that individuals with diabetes are at an increased risk of developing heart failure and there is evidence of increased risk even within the pre-diabetic range of blood glucose (74).

Microalbuminuria and other microvascular complications of diabetes are known to elevate the risks of heart failure (25). Results of the RENAAL trial by Zeeuw et al. indicate that patients with high baseline albuminuria (> or =3 g/g creatinine) had a 1.92-fold (95% CI, 1.54 to 2.38) higher risk for the cardiovascular endpoint and a 2.70-fold (95% CI, 1.94 to 3.75) higher risk for heart failure compared with patients with low albuminuria (<1.5 g/g) (75). Furthermore, the Heart Outcomes Prevention Evaluation Study results indicated that any degree of albuminuria is a risk factor for CV events in individuals with or without DM (76). As per the results of this study, a urine albumin creatine ratio > 17.7 mg/g was associated with a significant increase in the relative risk of hospitalization for heart failure [3.23 (95% CI, 2.54–4.10)] (76). Furthermore, each 3.5-mg/g increase in ACR increased the risk of hospitalization for heart failure by 10.6% (95% CI, 8.4–13.0%) (76). In the EMPA-REG OUTCOME trial, the presence of microvascular disease was associated with an increased risk of heart failure-related hospitalization (hazard ratio:1.63; 95% CI, 1.06–2.49; *P* = 0.025) (77).

While glycemic control seems to affect the relationships between diabetes and heart failure, it appears that the choice of specific anti-hyperglycemic agents must be made carefully. Nichols et al. note that Insulin alone or in combination with sulfonylureas could increase the risk of heart failure in people with diabetes (15). Furthermore, Roumie et al. in their report indicates that sulfonylurea use, compared with metformin use, was associated with a 43% increased risk of heart failure (adjusted hazard ratio: 1.43; 95% CI, 1.30–1.57) (78). Available evidence also indicates elevated risks of heart failure with thiazolidinediones, rosiglitazone, and pioglitazone (79). Results of the Prospective Pioglitazone Clinical trial in Macrovascular Events trial demonstrated an increase in the risk of hospitalization for heart failure with pioglitazone (hazard ratio: 1.41; 95% CI, 1.10–1.80; *P* = 0.007) (80). Scirica et al. indicate that saxagliptin (a DPP-4 inhibitor) as compared to placebo was associated with an increased risk of hospitalization for heart failure among patients with T2D at high risk of cardiovascular outcomes (hazard ratio, 1.27; 95% CI, 1.07–1.52; *P* = 0.007) (81). Another study by Zannad et al. noted that alogliptin as compared to placebo demonstrated a nonsignificant increase in the risk of hospitalization for heart failure (hazard ratio, 1.19 (95% CI, 0.90–1.58) (82).

Decline in eGFR is a strong predictor of Heart failure in people with type 1 and type 2 diabetes (83). Ninomiya et al. point out that the presence of diabetes increases the rate of heart failure related hospitalization and contributes to poor survival (84). Furthermore, several cardiovascular outcome trials observed an approximately 2-fold increase in the heart failure rate in people with T2D and a reduced eGFR (85). Specifically, survival decreased from 2.8 years at an eGFR of 45–59 mL/min/1.73 m2 to 0.7 years at an eGFR of < 15

mL/min/1.73 m2 (85). Lawson et al. in their study note that in people with new onset heart failure, hospitalizations and deaths are high in patients with T2D or CKD and worst in those with both comorbidities (85).

Obrezan *and Kulikov*, indicate that atrial fibrillation is one of the most common concomitant diseases in patients with diabetes mellitus (DM) (86). As Obrezan *and Kulikov*, explain meta-analyses of multiple studies have shown that the risk of atrial fibrillation is higher for diabetic patients with impaired glucose homeostasis than for patients without diabetes. Furthermore, this study explains that patients with atrial fibrillation and diabetes were younger and had a higher frequency of arterial hypertension, chronic kidney disease, heart failure, ischemic heart disease, and stroke (87).

Additional cardiovascular risk, particularly heart failure has been reported with enlargement of epicardial adipose tissue on imaging. Several observational studies have reported that patients with type-2 diabetes have an abnormally enlarged and biologically transformed epicardial adipose tissue (EAT) compared with non-diabetic controls (88). This expanded EAT along with causing mechanical constriction of the diastolic filling is also a source of pro-inflammatory mediators capable of causing inflammation, microcirculatory dysfunction and myocardial fibrosis (88). In addition to being a cardiovascular risk factor, EAT may guide the treatment choices as drugs such as metformin, glucagon-like peptide-1 (GLP-1) receptor agonists and sodium-glucose cotransporter 2 inhibitors (SGLT2i), have been associated with attenuation of EAT enlargement (88).

New England Journal has published a study with the: An analysis of the Framingham study utilizing BNP in primary prevention in a community setting 3346 without heart failure, plasma natriuretic peptide levels predicted the risk of death and cardiovascular events after adjustment for traditional risk factors. The excess risk was apparent at natriuretic peptide levels well below the current thresholds used to diagnose heart failure. Examination of the relations of plasma B-type natriuretic peptide and N- terminal pro–atrial natriuretic peptide to the risk of death from any cause, a first major cardiovascular event, heart failure, atrial fibrillation, stroke or transient ischemic attack, and coronary heart disease (89).

### 3.5 Biomarkers and cardiovascular risk detection in type 2 diabetes

Biomarkers play a central role in the diagnosis and treatments of diabetes mellitus. Along with their diagnostic utility, biochemical parameters such as plasma glucose or HbA1C guide treatment-related decisions, as well. Over the years, accruing evidence with novel biomarkers have opened up several vistas for incremental improvements in diagnosis, prognosis and risk stratification of patients with type 2 diabetes mellitus. The American Diabetes Association recommends measuring HbA1C twice every year in diabetic individuals attaining treatment goals (90). In patients not attaining treatments goals, the American Diabetes Association recommends HbA1c testing at least once in every three months (90). Furthermore, the American Diabetes Association recommends measurement of urinary albumin at least once a year for screening of chronic kidney disease (90). It is important to note that there is a linear relationship between Urine Albumin-Creatinine Ratio and outcomes (91). There is a remarkably increased risk in mortality and cardiovascular death even in those patients with mildly elevated concentrations between 10 mg/g and 30 mg/g and care must be taken with this subset of patients, since they are often may not be identified as having chronic kidney disease risk. The utility of eGFR in predicting cardiovascular outcomes has also been relatively well-studied. The relationship between eGFR and cardiovascular death and overall mortality is “U-shaped” with the lowest risk in those patients with eGFR approximately 90–100 mL/min/m2 (92). The risk increases substantially with a lower eGFR, but also increases with eGFR >105 mL/min/m2, likely representing the hyperfiltration seen with early diabetic nephropathy (6) (**Box 4**).

The heterogeneity of type 2 diabetes mellitus offers several challenges for assessing cardiovascular risks. This consideration is particularly relevant when assessing risk in T2DM patients without manifest cardiovascular disease and in those with a risk of ischemic complications, which varies along with age, duration of diabetes, and comorbidities.

The findings of PEGASUS sub-study suggest that a strategy incorporating hsTn testing into a guideline-derived ASCVD risk algorithm provides enhanced risk stratification and reclassifies patients into more appropriate risk groups. This application of hsTn might be used to optimize the care of patients with ASCVD (93). Using a cut point of great plan 6ng/L showed a two-fold increase risk of cardiovascular events (94, 95). In primary prevention, the prediction of higher-risk of atherosclerotic events would guide the clinician to prescribe high-intensity statin therapy and the use of aspirin based on elevated troponin levels This effect is particularly important in diabetics as was confirmed in the zodiac study (96–98). The Utilization of biomarkers and ECG will facilitate the primary care solution and diabetologists and nephrologists to identify the higher-risk patient who would benefit from additional cardiovascular assessment, in the case of asymptomatic patient with atrial fibrillation discovered by ECG; additional therapies are indicated including anticoagulation based on CHA2DS2-VASc score AND decision on rhythm control which ultimately requires additional investigation by the cardiologist at a minimum echocardiography is essential to ensure the absence of valvular or ischemic heart disease or heart failure. The cardiologist may also despite to perform more detailed ischemic evaluation as appropriately indicated. Another scenario that would likely be encountered in clinical practice by the general physician is the elevation of NT-proBNP with the current guideline recommended cut of point of 125, this biomarker suggests not only underlying heart failure but also the presence of elevated cardiovascular risk. From this perspective, additional therapies can be recommended by the primary care physician including ACE inhibitor or ARB and particularly the addition of SGLT-2 inhibitors. Moreover, fast track referral to the cardiologist is essential to determine the type of heart failure; whether reduced ejection fraction or preserved ejection fraction and additional imaging to assess the type of LV dysfunction would be obtained. Similarly, an elevated high-sensitivity troponin would indicate the presence of high risk of atherosclerosis and predict future cardiovascular events. From the general physician standpoint, this would indicate the need of intensification of lipid- lowering therapy and addition of anti-platelet therapy as well; however, referral to the cardiologist for ischemia evaluation would be strongly recommended. Notably in the UAE, given the predominant sedentary lifestyle and increased preponderant risk factors, including family history, smoking, obesity and metabolic syndrome the likelihood of effort-inducing symptoms of angina or ischemia or heart failure is reduced due to the central lifestyle, thus specific questions for history examination should highlight the true or absence of symptoms and also exercise testing or appropriate ischemia evaluation. Additionally, a more detailed symptom evaluation in old patients is necessary, particularly in females since ethe presentation of coronary disease symptoms in females is generally atypical and symptoms of palpitation, fatigue and dizziness epigastric and other atypical chest pain may be predominant and is often accredited to long cardiac diagnosis or assumed to be non-cardiac. Thus, the utilization of more precise stratification using biomarkers for initial assessment is strongly recommended. Additional cardiovascular examinations may also be recommended in selective patients to more precisely determine the risk. Ankle Brachial index is a simple clinic-based test that can easily discriminate the presence of early stages of peripheral atherosclerosis, also referral for coronary CT calcium score is recommended in the guidelines to reclassify cardiovascular risk and recommend preventive therapy, particularly statins (8, 99).

Available data indicates that increased concentrations of natriuretic peptides in patients with type 2 diabetes are associated with increased cardiovascular risk and have a good value for predicting cardiovascular mortality and hospitalization for heart failure. Natriuretic peptides are excellent discriminators of risk and their utilization to guide the additional therapy, particularly SGLT-2 inhibitors, in order to reduce heart failure in type 2 diabetes at the earliest possible stages (96).

The ADA consensus highlights that the detection of people at high risk for HF (stage A) or those with stage B Heart failure would facilitate early interventions to avert the progression of heart failure to advanced stages (1). Furthermore, screening for heart failure in asymptomatic patients can help in replacing diabetes medications that increase the risk of heart failure with diabetes medications such as SGLT2 inhibitors, which are known to improve heart failure outcomes. While the utility of biomarkers for heart failure screening in people with diabetes was met with limited adoption, the advent of extensive and robust evidence strongly recommends the routine utilization of natriuretic peptides for detecting heart failure risks in people with diabetes. Huelsmann et al. in their study highlight that the negative predictive value of a normal value (<125 pg/mL) of NT-proBNP for short-term cardiovascular events in diabetic patients is about 98% (100).

Furthermore, the results of the PONTIAC trial demonstrate the utility of NT-proBNP in guiding referrals to cardiac clinics. In this study, hospitalization/death due to cardiac disease was reduced by 65% after two years in patients with elevated NT-proBNP (>125 pg/ml), who were referred to additional care at a cardiac outpatient clinic for the up-titration of the renin-angiotensin system antagonists and beta-blockers (101). Similar findings have been reported by the STOP-HF trial, wherein referrals to echocardiogram and cardiology clinics based on BNP testing resulted in significant reductions of LV dysfunction with or without heart failure (45%), heart failure (52%), and emergency hospitalization for major cardiovascular events (55%) (102). Overall, it appears that BNP or NT-proBNP–based screening followed by team-based care, including a cardiovascular specialist, can be useful to prevent the development of LV dysfunction or new-onset heart failure (6).

*Alexandre Mebazaa et al.* in the STRONG-HF trial aimed to address under-treatment in AHF patients with rapid up-titration of GDMT compared with usual standard-of-care.

Rapid up-titration of GDMT under close follow-up (physical examination biomarkers including NT-proBNP) during and soon after discharge from HF hospital admission is safe, with no increase in serious adverse events versus usual care.

The STRONG-HF treatment regimen resulted in a significant 34% risk reduction of HF readmission and all-cause mortality at 180 days.

Both BNP and NT-BNP are widely available and Reimbursed by Insurance Payors in UAE. That’s why, rapid action is needed to implement the STRONG-HF management regimen into daily clinical practice.

### 3.6 Utilization of natriuretic peptides in patients with CKD

In those with reduced renal function, NT-proBNP can be increased because it is excreted from kidney. However, they are still valuable for cardiovascular screening for several reasons. These elevated levels of NT-proBNP in patients with CKD do not simply reflect the reduced clearance of the peptide; rather, they largely reflect a true-positive finding, identifying the presence of heart disease in these patients, while similarly indicating prognosis as well. Christopher DeFilippi et al. (https://pubmed.ncbi.nlm.nih.gov/18243865/.

Van Kimmenade et al. (https://pubmed.ncbi.nlm.nih.gov/19264247/) demonstrated that renal extraction of both BNP and NT-proBNP changed minimally across a spectrum of renal function measured down to approximately an eGFR of 30 mL · min−1 ·.

Importantly, when using NT-proBNP to evaluate a patient with dyspnea and impaired renal function, the recommended cut points of 450, 900, and 1,800 ng/L for those aged 75 years, respectively, do not require further adjustment for renal function. Thus, NT-proBNP testing remains useful for the diagnostic and prognostic evaluation of patients with CKD”.

In this >11K participants in a general population study (https://pubmed.ncbi.nlm.nih.gov/37290699/) showed that “Despite its strong inverse association with eGFR, NT-proBNP has robust associations with mortality across the full range of kidney function in the general US adult population.”

### 3.7 Cost effectiveness of natriuretic peptide in HF diagnosis and risk assessment in diabetic patients

The trials (Gallagher et al. paper) have proven that natriuretic peptide-based screening and targeted prevention can reduce heart failure and left ventricular dysfunction and other major cardiovascular events.

This approach is now part of North American guidelines and European Guidelines Walter et al. (103). Cost-effectiveness of NT-proBNP in Diagnosis of heart failure in high-risk patients especially Diabetic Patients on the health care system in terms of reducing the burden on the patient journey, and the healthcare system. This is very important given the UAE insurance restrictions on risk assessment and screening. Many studies have shown the budget impact and cost-effectiveness of pro-BNP and this has been universally shown to result in cost savings through reduction of medical visits, hospitalizations, and unnecessary echo-cardiographic testing. In addition, clinical trials have shown that natriuretic peptide screening and targeted preventive therapies can reduce heart failure and other major cardiovascular events. This approach is now incorporated in the ADA consensus guidelines. It is well-recognized that the earlier diagnosis using biomarkers will allow effective therapeutic preventions that prevent the incidence of heart failure in the first place and prevent progression to overt symptomatic LV dysfunction. It is also recognized the cost of acute hospitalization and inpatient care far exceeds the cost of outpatient natriuretic peptide and medical therapy (103).

**Figure 2:**
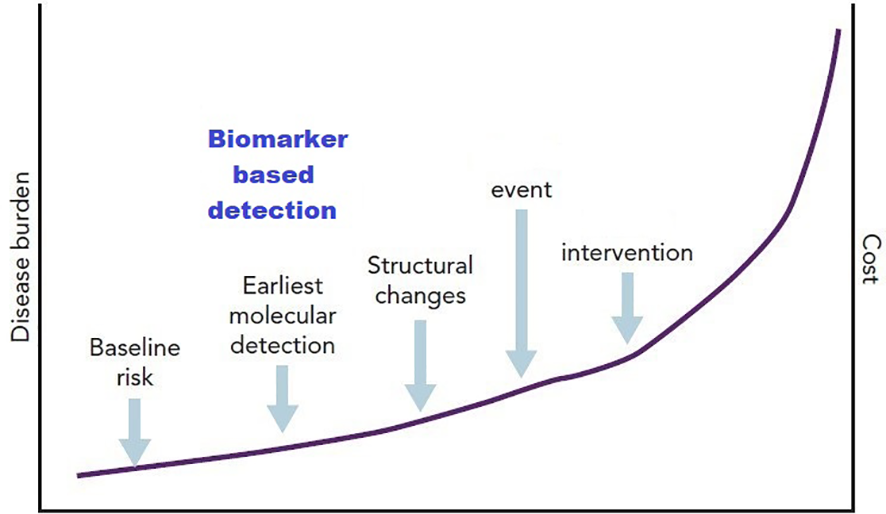
Diagrammatic representation of the concept of biomarkers as a component of stage B heart failure (104).

The diagram below shows that biomarker-based detection has a role in the earlier molecular detection in which the cost is low if compared with the cost in a clinical event, in other words the earlier detection may have a cost-effective value (**Figure 2**).

Gallagher et al., paper summarizing the 2 landmark Trials PONTIAC 1 and STOP-HF stating the use of NT-pro BNP is an effective tool in refining risk prediction for heart failure and cardiovascular disease and adding predictive power to conventional risk factors (104). A subsequent analysis of the cost-effectiveness of this approach was undertaken. The cost per quality- adjusted life year gain was €1,104 and the intervention has an 88% probability of being cost- effective at a willingness to pay threshold of €30,000 (89).

### 3.8 Cost-effectiveness of natriuretic peptide-based screening and collaborative care

A report from the STOP-HF (St Vincent’s Screening TO Prevent Heart Failure) study Ledwidge et al. (105), assessed the cost-effectiveness of natriuretic peptide-based screening in a sub study of 1054 participants with cardiovascular risk factors (about 18% with diabetes mellitus) found cardiovascular hospitalization savings offset increased outpatient and primary care costs. The cost per case of LVD/HF prevented was €9683 (sensitivity range –€843 to €20 210), whereas the cost per MACE prevented was €3471 (sensitivity range –€302 to €7245), in addition, the cost per QALY gain was €1104 and the intervention has an 88% probability of being cost-effective at a willingness to pay threshold of €30 000. There were 157 deaths and/or emergency hospitalizations for major adverse cardiac events (MACE) in the control group vs. 102 in the intervention group (OR 0.68; 95% CI 0.49–0.93; P = 0.01). Conclusion: Among patients with cardiovascular risk factors, natriuretic peptide-based screening and collaborative care reduced LVD, HF, and MACE, and has a high probability of being cost-effective.

A recent simulation study from Austria and Switzerland, a cost–utility model was developed to simulate the cost-effectiveness of NT-proBNP-supported screening of undetected HF with and without Diabetes as well as its long-term consequences in HF population in patients from age 60 over lifetime (103). In this study, the per-patient incremental cost–utility ratio (ICUR)/QALY of NT-proBNP vs. no screening was €3,042 and CHF 897 for HF patients in Austria and Switzerland respectively. This study concludes that screening with NT-proBNP biomarkers is a highly cost-effective or cost-saving diagnostic option for patients with HF, and a sensitivity analysis confirmed these findings.

The Irish Government in conjunction with Irish Heart Foundation conducted systemic review to assess the impact of early diagnosis if Heart Failure in the context of patient and healthcare system factors with a view to reduce the cost and frequency of heart Failure Hospitalization concluding the significant importance of early diagnosis and how it will positively impact the financial outcomes.

A survey of 372 heart failure patients in Ireland, found 60% of patients surveyed in this study received their diagnosis from a heart specialist in this hospital but with a reported 14 month waiting time to see a specialist.

Delayed diagnosis was found to have a profound impact on costs, with a 6-month delay leading to a 23% increase in emergency hospitalizations and contributing to an increased number of bed days.

This has resulted in the introduction of the Enhanced Community Care (ECC) Program, which, since 2021, has been facilitating a phased direct access to NT-proBNP blood testing for GPs for the full adult population. This will support GPs to triage patients with heart failure for referral, which as part of the Structured Chronic Disease Management Programme (CDM), will allow GPs to better prevent and manage chronic diseases, including heart failure (106). Heart failure policy and practice in Europe: Ireland (107).

Antonio Leon-Justel et al., showed that by using combined biomarkers of NT-pro BNP and high sensitivity Troponin T led to significant reduction in total hospitalization rate by 19%, and length of stay by 7 days and frequency of ED visits by 44%. The overall cost saving associated with the intervention was € 72,769 per patient (from € 201,189 to € 128,420) and €139,717.65 for the whole group over 1 year. Suggesting that greater attention should be given to this high-risk cohort to minimize the risk of hospitalization readmissions (108).

Irish Heart Foundation has published a report about the “ State of Heart ‘‘ initiative and highlighting in which the utilization of NP screening in general practice with highlighted particularly in terms of its benefit in early diagnosis of heart failure and its positive impact on patient and healthcare system. This has resulted in the introduction of the Enhanced Community Care (ECC) Program, which, since 2021, has been facilitating a phased direct access to NT-proBNP blood testing for GPs for the full adult population. This will support GPs to triage patients with heart failure for referral, which as part of the Structured Chronic Disease Management Programme (CDM), will allow GPs to better prevent and manage chronic diseases, including heart failure.

A further analysis from Leon-Justel et al. study from Spain about “Biomarkers-based personalized follow-up in chronic heart failure improves patient’s outcomes and reduces care associate cost” A cost analysis was also performed on these data. Therefore, a personalized follow-up of HF patients led to important outcome benefits and resulted in cost savings, mainly due to the reduction of patient hospitalization readmissions and a significant reduction of care-associated costs, suggesting that greater attention should be given to this high-risk cohort to minimize the risk of hospitalization readmissions (108).

## 4 Managing cardio-metabolic and renal risk in diabetes

Managing the cardio-renal risk in diabetes requires an integrated multi-disciplinary approach involving general practitioners, nurses, diabetes educators, endocrinologists, cardiologists and nephrologists. Proper attention to diet and physical activity is imperative. Lowering the risk of myocardial infarction, heart failure, stroke, amputation and decline in kidney functions will need control of blood pressure, blood sugar, dyslipidemia in addition to managing the heightened thrombotic risk.

### 4.1 Managing hypertension

**Figure 3:**
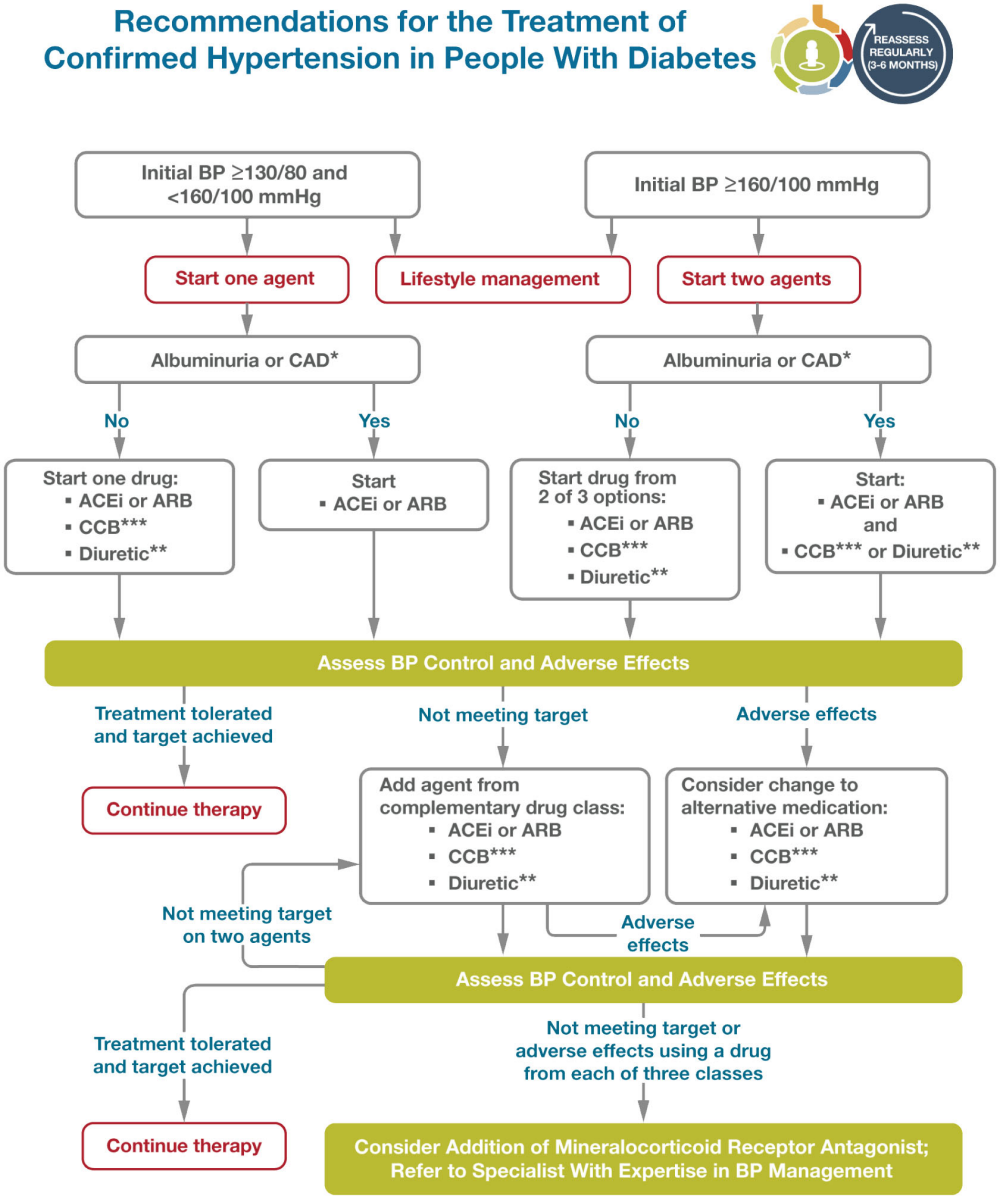
Reprinted with permission from / Adapted with permission from 10. Cardiovascular Disease and Risk Management: Standards of Care in Diabetes— 2024 by American Diabetes Association Professional Practice Committee; ElSayed, Nuha A., licensed under 5903060036974, American Diabetes Association.

**Figure 3** depicts an algorithm for treatment of hypertension in people with diabetes. Adapted from Elsayed NA. (109). Several studies have demonstrated the usefulness of antihypertensive therapy in reducing atherosclerotic cardiovascular disease, heart failure, and microvascular complications in diabetes. Screening for and treating hypertension must be an integral part of the holistic treatment of diabetic patients (110, 111). The treatment of hypertension to blood pressure goals below 130/80 mm Hg is the recommended treatment goal, with drug therapies considered for people with diabetes and hypertension.

According to the recent update of the ADA 2023 standard of care has changed the recommendation on blood pressure treatment goals in individuals with diabetes, this was revised to target a blood pressure of <130/80 mmHg. In particular, the recently reported results of the STEP (Strategy of Blood Pressure Intervention in the Elderly Hypertensive Patients) trial were added. In addition, the guideline was updated to consider pharmacological treatment in people with diabetes and a confirmed blood pressure ≥130/80 (98).

### 4.2 Managing hyperglycemia

**Figure 4:**
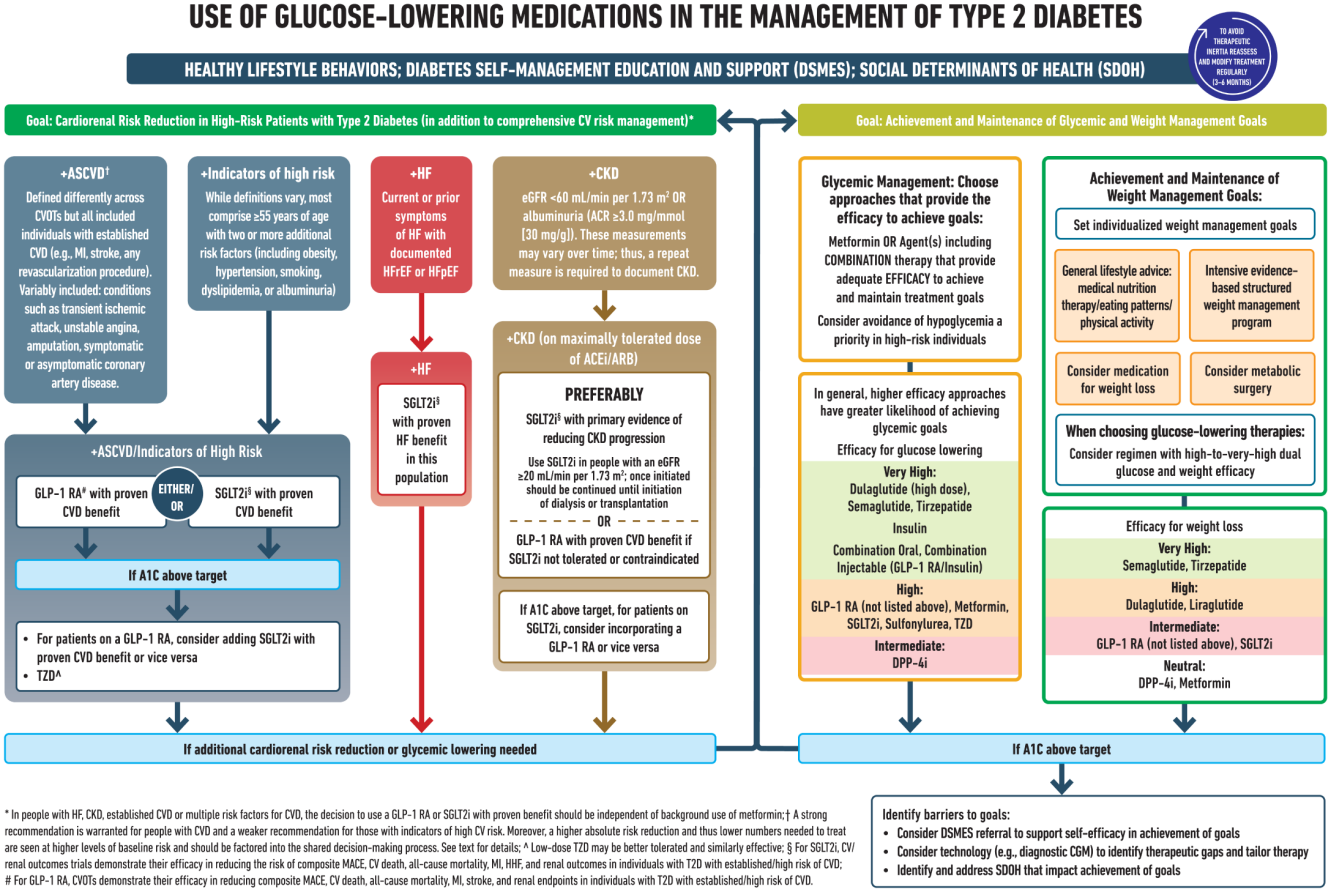
Reprinted with permission from / Adapted with permission from 9. Pharmacologic Approaches to Glycemic Treatment: Standards of Care in Diabetes— 2023 by ElSayed, Nuha A.; Aleppo, Grazia, licensed under 5903051181630, American Diabetes Association.

Lifestyle modifications are key adjuncts to diabetes therapy and should be emphasized at all stages in the management of patients with diabetes mellitus (112). Adapted from Elsayed NA. (113) (**Figure 4**).

Furthermore, results of several recent cardiovascular outcome trials (CVOTs) indicate that SGLT2i and GLP1RA, along with lower glucose levels also afford clinically meaningful cardio-renal benefits (114, 115). The current guidelines heavily prioritize the early initiation and utilization of cardio-protective and renal-protective diabetes medications particularly SGLT2 and GLP1 irrespective of HBA1C (even if HBA1C is controlled) in particular, this would apply to patients who are recognized to have early stage heart failure during screening with natriuretic peptides.

Piopioglitazone is another agent with cardiovascular benefits, especially in stroke patients; however must not be used in patients with heart failure, i.e natriuretic peptide screening may identify patient’s ineligible for Pioglitazone (81, 116). In addition, saxagliptin is a black box warning for heart failure and should only be used with appropriate heart failure-related caution again natriuretic peptides can help the clinician identify patients contra-indicated with saxagliptin (117, 118). Once the cardiovascular, CKD, or HF conditions have been addressed, anti-hyperglycemic regimens aimed at glucose reduction should be implemented to meet glycemic goals for the individual patient (119).

Individualized goals for HbA1c and other glucose measures should be considered. As stipulated in recommendations from AACE and ADA, HbA1c goal between 6.5% and 7.0% is appropriate for most patients. Younger, healthier patients at lower cardiovascular risk may benefit from HbA1c goals closer to normal (<6.0%), whereas higher A1C goals (~7.5% or higher) may be appropriate for older patients with more complex diseases complicated by multiple comorbidities.

Combination therapy with agents having complementary mechanisms of action must be considered to reduce clinical inertia in patients whose HbA1c is >1- 2% above their individualized goal, even if such patients have newly diagnosed T2D. Incretin classes (GLP1-RAs and dipeptidyl peptidase 4 (DPP4) inhibitors should not be combined with each other. Although insulin is associated with weight gain and the risk of hypoglycemia, it should not be withheld from patients who cannot meet their glucose goals using other agents, and insulin should be used in any patient exhibiting symptoms of uncontrolled diabetes.

### 4.3 Managing dyslipidemia

It is advisable to perform a lipid profile at the time of diagnosis or initial evaluation of diabetes and at least every 5 years thereafter in patients under the age of 40 years. A lipid panel should also be obtained immediately before initiating statin therapy and 4–12 weeks after. Further testing should be done after dose changes and periodically on an individual basis to screen for adherence.

**Figure 5:**
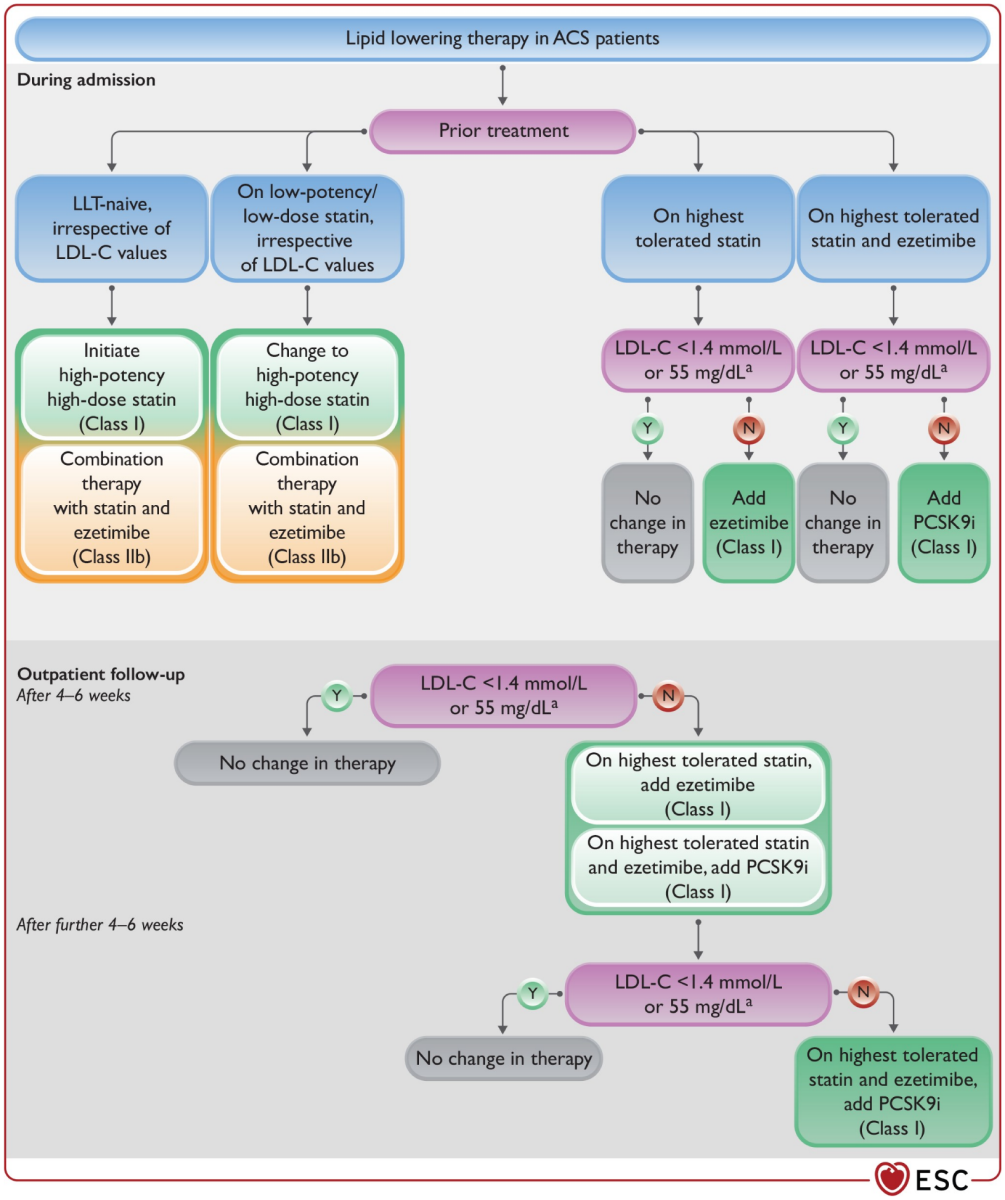
Lipid-lowering therapy in ACS patients. ACS, acute coronary syndrome; LDL-C, low- density lipoprotein (107).

Atherosclerotic cardiovascular disease risk is variable among individuals with diabetes and is influenced by traditional risk factors and risk modifiers specific to diabetes (117). A clinician’s global assessment of risk can help identify the benefits of an increase in statin therapy. Risk-enhancing factors that should be taken into consideration in patients with dyslipidemia and diabetes are listed in **Box 5**. Adapted from Byrne RA. (107) (**Figure 5**).

Box 5 **Diabetes-specific risk enhancing factors.**

• Long duration (≥10 years for T2DM, ≥20 years for T1DM)
•Albuminuria ≥30 mcg of albumin/mg creatinine
• eGFR <60 mL/min/1.73 m3
• Retinopathy
• Neuropathy
○ ABI<0.9

### 4.4 Managing the thrombotic risk

The benefit of using aspirin in primary prevention remains controversial (120, 121). Data from randomized controlled trials of aspirin in diabetics failed to show a significant reduction in overall ASCVD end points (122, 123). A meta- analysis by Baigent et al., showed that aspirin reduces the risk of serious vascular events by 12% (relative risk 0.88 [95% CI 0.82–0.94]) (124). In this study, the most significant reduction was for nonfatal MI, with little effect on cardiovascular mortality (relative risk 0.95 [95% CI 0.78–1.15]) or total stroke (124). Furthermore, results of the ASCEND (A Study of Cardiovascular Events in Diabetes) trial indicates that aspirin was associated with major bleeding (125). Aspirin appears to have a modest effect on ischemic vascular events, with the absolute decrease in events depending on the underlying ASCVD risk (126).

Pregnant individuals with type 1 or type 2 diabetes should be prescribed low-dose aspirin 100–150 mg/day starting at 12 to 16 weeks of gestation to lower the risk of preeclampsia. Use aspirin therapy (75–162 mg/day) as a secondary prevention strategy in those with diabetes and a history of atherosclerotic cardiovascular disease. Combination therapy with aspirin plus low-dose rivaroxaban should be considered for individuals with stable coronary and/or peripheral artery disease and low bleeding risk to prevent major adverse limb and cardiovascular events. Aspirin is not recommended for those at low risk of ASCVD (such as men and women aged <50 years with diabetes with no other major ASCVD risk factors) as the low benefit is likely to be outweighed by the risks of bleeding.

Clinical judgment should be used for those at intermediate risk (younger patients with one or more risk factors or older patients with no risk factors) until further research is available (96, 98).

Aspirin is recommended for primary prevention in men and women aged ≥ 50 years with diabetes and at least one additional major risk factor such as family history of premature ASCVD, hypertension, dyslipidemia, smoking, or chronic kidney disease/albuminuria and who are not at increased risk of bleeding (127–129).

Furthermore, use of noninvasive imaging techniques such as coronary calcium scoring may potentially help further tailor aspirin therapy, particularly in those at low risk. For patients above the age of 70 years (with or without diabetes), the risk of bleeding with aspirin outweighs it’s benefit and is not recommended (123, 125, 130).

Further details on the management of ischemic heart and vascular diseases, including the selection of antianginal agents, indications for revascularization, rehabilitation protocols, and the specific management of heart failure, coronary artery disease, and peripheral vascular disease, are beyond the scope of this document. For comprehensive guidance on these topics, readers are directed to the relevant society guidelines on the management of these conditions.

### 4.5 Managing cardio-renal risk in diabetes

The management of diabetes requires a comprehensive approach that addresses both macrovascular and microvascular complications to reduce the overall burden of the disease. It is imperative to implement strategies that effectively mitigate these complications, as they are significant contributors to morbidity and mortality in diabetic patients. The selection of anti-diabetic agents should be made with careful consideration of the patient’s cardiovascular risk profile. In particular, the use of SGLT2 inhibitors and long-acting GLP1 receptor agonists (GLP1-RAs) is strongly recommended for individuals with an elevated risk of atherosclerotic cardiovascular disease (ASCVD), chronic kidney disease (CKD), and/or heart failure. These agents have demonstrated significant benefits in reducing cardiovascular events and slowing the progression of kidney disease in high-risk populations.

Furthermore, for primary prevention of cardiovascular events, the use of aspirin is advised in men and women aged 50 years or older who have diabetes and at least one additional major risk factor, such as a family history of premature ASCVD, hypertension, dyslipidemia, smoking, or chronic kidney disease/albuminuria, provided they are not at an increased risk of bleeding. This recommendation underscores the importance of individualized risk assessment in the management of diabetic patients.

In addition to these pharmacologic interventions, the use of renin-angiotensin-aldosterone system (RAAS) blockers, particularly angiotensin-converting enzyme inhibitors (ACE inhibitors) or angiotensin II receptor blockers (ARBs), is indicated in diabetic patients to reduce cardiovascular and renal risks. The recent inclusion of finerenone, a non-steroidal mineralocorticoid receptor antagonist, further enhances the therapeutic options available for preventing cardiorenal complications in diabetes mellitus. By incorporating these strategies, healthcare providers can more effectively manage the complex interplay between diabetes and cardiovascular disease, ultimately improving patient outcomes.

These recommendations are summarized in **Box 6**.

Box 6 **Consensus statement – Managing Cardio-renal Risk in Diabetes.**

• Strategies to mitigate both macrovascular and microvascular complications of diabetes are recommended.
• Choice of anti-diabetic agents must be made in a way so as to reduce the burden of cardiovascular risk.
• SGLT2 inhibitors and long-acting GLP1-RAs are recommended for people with elevated risk of atherosclerotic cardiovascular disease, chronic kidney disease and/or heart failure.
• Aspirin is recommended for primary prevention in men and women aged ≥ 50 years with diabetes and at least one additional major risk factor such as family history of premature ASCVD, hypertension, dyslipidemia, smoking, or chronic kidney disease/albuminuria and who are not at increased risk of bleeding.
• RAAS blockers ACE/ARB are indicated in particular.
 ○ Finerenone is also recommended for prevention of cardiorenal risk in DM.

## 5 Conclusion

The combination of diabetes and cardiovascular disease, particularly undiagnosed heart failure persists as a major burden in United Arab Emirates. Several modifiable and non-modifiable risk factors such as advanced age, long-standing duration of diabetes, poor glycemic control, CKD and common albuminuria with presence of co-morbidities are predictors of heart failure. In people with these risk factors, it is very important to recommend routine measurement (at least yearly) of natriuretic peptides before referring to imaging tests and in selected patients high-sensitivity troponins can also be performed. This approach will identify patients with early stages of cardiovascular disease and heart failure and guide treatment early with cardio-protective therapy.

With respect to treatments, standards of care must include SGLT2 inhibitors combined with RAAS blockers for heart failure stages B, C and D and GLP-1 receptor agonist with metformin for atherosclerotic cardiovascular disease or peripheral artery disease. While insulin DPP-4 and/or metformin can be useful for additional glycemic control and thiazolidinediones must be avoided in heart failure stages B, C and D.
